# “You produce while I clean up”, a strategy revealed by exoproteomics during *Synechococcus*–*Roseobacter* interactions

**DOI:** 10.1002/pmic.201400562

**Published:** 2015-04-21

**Authors:** Joseph A. Christie‐Oleza, David J. Scanlan, Jean Armengaud

**Affiliations:** ^1^ School of Life SciencesUniversity of WarwickCoventryUK; ^2^ CEADSV, IBiTec‐S, SPI, Li2D, Laboratory “Technological Innovations for Detection and Diagnostic”Bagnols‐sur‐CèzeFrance

**Keywords:** Dissolved organic matter, Exoproteome, Marine microbial interactions, Microbiology, *Roseobacter*, Secreted enzymes

## Abstract

Most of the energy that is introduced into the oceans by photosynthetic primary producers is in the form of organic matter that then sustains the rest of the food web, from micro to macro‐organisms. However, it is the interactions between phototrophs and heterotrophs that are vital to maintaining the nutrient balance of marine microbiomes that ultimately feed these higher trophic levels. The primary produced organic matter is mostly remineralized by heterotrophic microorganisms but, because most of the oceanic dissolved organic matter is in the form of biopolymers, and microbial membrane transport systems operate with molecules <0.6 kDa, it must be hydrolyzed outside the cell before a microorganism can acquire it. As a *simili* of the marine microbiome, we analyzed, using state‐of‐the‐art proteomics, the exoproteomes obtained from synthetic communities combining specific *Roseobacter* (*Ruegeria pomeroyi* DSS‐3, *Roseobacter denitrificans* OCh114, and *Dinoroseobacter shibae* DFL‐12) and *Synechococcus* strains (WH7803 and WH8102). This approach identified the repertoire of hydrolytic enzymes secreted by *Roseobacter*, opening up the black box of heterotrophic transformation/remineralization of biopolymers generated by marine phytoplankton. As well as highlighting interesting exoenzymes this strategy also allowed us to infer clues on the molecular basis of niche partitioning.

**Abbreviations**
CDS: coding domain sequences
DOM: dissolved organic matter
GTA: gene transfer agent
NSAF: normalized spectral count abundance factor

## 1 Introduction

Oceans are by far the largest ecosystems on Earth. These aquatic systems, covering over 70% of the World's surface, comprise 1.3 × 10^9^ cubic kilometers that are largely populated by organisms living in the water column, known as plankton. These organisms are mostly in the microscopic range (0.2–2 μm, named picoplankton) and mainly encompass bacteria and picoeukaryotes. Hence, the marine microbiome is the largest microbial system known and has enormous influence on global processes such as climate and biogeochemical cycles. In contrast to terrestrial ecosystems, marine food webs are mainly sustained by microbial photosynthetic primary producers or phytoplankton 1 as they are the main source of carbon and energy that fuel the ecosystem despite their relatively low numerical abundance and contribution to biomass (under 10% of total plankton) 2, 3, 4. Phytoplankton, due to leakage, inefficient grazing or viral lysis, can release large amounts of dissolved organic matter (DOM) and particulate organic matter that can then be used by the numerous heterotrophic microorganisms present in the water column 5, 6, 7. Heterotrophs will remineralize most of this primary produced organic matter, recycling essential elements like nitrogen, phosphorus, and trace‐metals within this nutrient‐poor ecosystem 8, 9, thereby determining the nature and quantity of carbon and nutrients that sink to the deeper layers of the ocean.

Most of the oceanic DOM is in the form of biopolymers that must be hydrolyzed outside the cell before a microorganism can acquire them, because transport systems operate with molecules smaller than 0.6 kDa 10. Hence, exoenzymes or ectoenzymes (those attached to the bacterial cell envelope) play an essential role in biopolymer hydrolysis and initial processing 11, 12. There have been several attempts to characterize enzyme activities in natural marine systems by use of fluorescently labeled compounds (e.g. see 12, 13, 14) but unfortunately most of the enzymes that carry out these vital functions remain to date unidentified. Microbial secretion of proteins is a universal phenomenon that allows microbes to modify or otherwise influence their community and environment. Therefore, we can infer an organism's ecological strategy and its function within the environment by analyzing its secreted proteins 15. Exoproteomics is the large‐scale study of extracellular proteins of a biological system. Interestingly, up to 35% of bacterially encoded genes are predicted to encode proteins secreted from the cell, including membrane‐linked proteins (e.g. membrane transporters, ectoenzymes, or motility proteins) that can easily be lost by the cell and found in the exoproteomic fraction 16. The exoproteomes of human pathogens and soil microorganisms such as fungi have been characterized to some extent, but those of free‐living marine organisms remain largely untapped.

Proteomic analyses of cell fractions of two different *Roseobacter* strains (an abundant and widespread group of marine heterotrophs) has previously been reported, focusing on *Ruegeria pomeroyi* DSS‐3 and *Phaeobacter inhibens* DSM 17395 17, 18, 19. We previously documented the first exoproteomes of the *Roseobacter* clade, showing that a large fraction of their exoproteome is involved in active membrane transport for scavenging utilizable sources of carbon and energy, but motility and toxin‐like proteins were also detected in abundance 15, 20, 21. In these experiments, *Roseobacter* cells were grown on “easily assimilated” substrates (i.e. succinate or hydrolyzed polypeptides). Recent work on the exoproteomes of the picocyanobacteria *Synechococcus*, one of the major marine primary producers in the oceans 4, showed an elevated leakiness of cytoplasmic polypeptides that could well support heterotrophic growth 22. Despite living in nutrient‐depleted environments, picocyanobacteria have proven to be leaky organisms per se, whether by cell lysis or via the release of numerous extracellular vesicles 23. The leaked cytoplasmic polypeptide fraction was strongly reduced when *Synechococcus* was co‐cultured with a heterotroph (i.e. *R. pomeroyi* DSS‐3) and, moreover, the presence of *Synechococcus* had an inducing effect on the exoproteolytic activity of the *Roseobacter* strain 22.

The main aim of this work was to document the repertoire of exoenzymes produced by *Roseobacter* strains that break down large‐size biopolymers produced by the naturally co‐occurring marine photoautotroph *Synechococcus* for its own assimilation. We first analyzed the exoproteome of *R. pomeroyi* DSS‐3 when grown with two different *Synechococcus* strains WH7803 and WH8102. Secondly, we compared the exoproteomes of three different *Roseobacter* strains in the presence of *Synechococcus* sp. WH7803. These synthetic systems, mimicking natural marine communities, revealed an interesting array of extracellular enzymes that provide new insights into the relevant biological processing of DOM by the marine microbiome.

## 2 Materials and methods

### 2.1 Bacterial strains and growth conditions

Marine *Synechococcus* strains WH7803 and WH8102 were grown in ASW medium 24 at 22ºC, 140 rpm, with a light intensity of 10 μmol photons m^−2^ s^−1^. The three *Roseobacter* strains *Ruegeria pomeroyi* DSS‐3, *Roseobacter denitrificans* OCh114, and *Dinoroseobacter shibae* DFL‐12 were routinely grown in marine broth (Difco) at 28ºC. *Roseobacter* cultures were grown to early‐stationary phase and washed three times in ASW prior to co‐inoculation with *Synechococcus*. *Roseobacter*–*Synechococcus* co‐cultures were grown in optimal conditions for the photoautotroph as described above. In experiment 1 we co‐cultured *Ruegeria pomeroyi* DSS‐3 with two different *Synechococcus* strains WH7803 and WH8102, whereas in experiment 2 we co‐cultured the three different *Roseobacter* strains with *Synechococcus* sp. WH7803 (Table 1). Cell counts of *Synechococcus* were performed by flow cytometry (BD FACScan) counting a minimum of 1000 cells. *Roseobacter* strains were counted by colony forming units on marine agar (Difco). Three biological replicates were performed for all experiments. Incubation times and cell counts at inoculum and harvest of each co‐culture experiment 1 and 2 is indicated in Table 1. To monitor the production of PaxA (an abundantly detected RTX‐like toxin, see 20), *R. pomeroyi* DSS‐3 was also grown in ASW and autoclaved seawater (Sigma) plus supplements.

**Table 1:** Cell counts from each co‐culture experiment from which the exoproteome was analyzed

	Co‐culture	Inoculum a ^),^ b ^)^	Harvest a ^),^ b ^)^	Incubation
Exp. 1	*Synechococccus* sp WH7803 *R. pomeroyi* DSS‐3	1 × 10^7^ 1 × 10^6^	1 × 10^8^ 2 × 10^7^	7 days
	*Synechococccus* sp WH8102 *R. pomeroyi* DSS‐3	3 × 10^7^ 1 × 10^6^	1 × 10^8^ 1 × 10^7^	
Exp. 2	*Synechococccus* sp WH7803 *R. pomeroyi* DSS‐3	3 × 10^6^ 1 × 10^7^	1 × 10^7^ 7 × 10^7^	3 days
	*Synechococccus* sp WH7803 *R. denitrificans* OCh114	3 × 10^6^ 5 × 10^7^	1 × 10^7^ 1 × 10^8^	
	*Synechococccus* sp WH7803 *D. shibae* DFL‐12	3 × 10^6^ 3 × 10^7^	1 × 10^7^ 7 × 10^7^	

a *Synechococcus* counts are fluorescent cells m/L. *Roseobacter* counts are colony forming units m/L.

b All counts are an average of three biological replicates. In all cases SD remained below 0.1‐fold.

### 2.2 Preparation of exoproteomes, trypsin in‐gel proteolysis, and nano‐LC‐MS/MS analysis

The milieu containing the secreted proteins of the different co‐cultures was obtained after removing the cells by centrifugation (3000 *g* for 15 min at room temperature) and subsequent gentle filtration through 0.22 μm pore size filter units (Sterivex‐GV, Millipore). Proteins in the remaining milieu were concentrated and purified by precipitation with trichloroacetic acid and separated using SDS‐PAGE as previously described 20. The equivalent of 40 mL of milieu (concentrated in 20 μL) was loaded onto a 10% Tris‐Bis NuPAGE gel (Invitrogen) and SDS‐PAGE was carried out using 1X MOPS solution (Invitrogen) as the running buffer. Exoproteomes were run in short gel migrations (3 mm) for subsequent shotgun proteomics analysis. Polyacrylamide gel bands containing the entire exoproteome were cut and processed for in‐gel proteolysis with trypsin (Roche) as previously described 25. Nano‐LC‐MS/MS experiments for experiment 1 (Table 1) were performed using an LTQ‐Orbitrap XL hybrid mass spectrometer (ThermoFisher) coupled to an UltiMate 3000 LC system (Dionex‐LC Packings) using conditions previously described 26. Nano‐LC‐MS/MS experiments for experiment 2 (Table 1) were performed with an Orbitrap Fusion mass spectrometer to render a higher coverage of the exoproteomes. Conditions used were as follows: two columns were utilized to separate tryptic peptides by reverse phase chromatography, an Acclaim PepMap μ‐precolumn cartridge 300 μm id × 5 mm 5 μm 100 Å and an Acclaim PepMap RSLC 75 μm × 50 cm 2 μm 100 Å (Thermo Scientific). The columns were installed on an Ultimate 3000 RSLCnano system (Dionex). Mobile phase buffer A comprised 0.1% v/v aqueous formic acid and mobile phase B comprised 80% v/v acetonitrile containing 0.1% v/v formic acid. Samples were loaded onto the μ‐precolumn equilibrated in 2% v/v aqueous acetonitrile containing 0.1% v/v trifluoroacetic acid for 8 min at 10 μL/min after which peptides were eluted onto the analytical column at 250 nL/min by increasing the mobile phase B concentration from 3% B to 35% over 27 min then to 90% B over 5 min, followed by a 4 min wash at 90% B and a 12 min reequilibration at 3% B. Eluting peptides were converted to gas‐phase ions by means of electrospray ionization and analyzed on a Thermo Orbitrap Fusion mass spectrometer (Q‐OT‐qIT, Thermo Scientific). Survey scans of peptide precursors from 350 to 1500 *m/z* were performed at 120 K resolution (at 200 *m/z*) with a 4 × 10^5^ ion count target. Tandem MS was performed by isolation at 1.6 Th using the quadrupole, HCD fragmentation with normalized collision energy of 35, and rapid scan MS analysis in the ion trap. The MS/MS ion count target was set to 104 and the max injection time was 200 ms. Precursors with charge state 2–4 were selected and sampled for MS/MS. The dynamic exclusion duration was set to 45 s with a 10 ppm tolerance for the selected precursor and its isotopes. Monoisotopic precursor selection was turned on. The instrument was run in top speed mode with 2 s cycles. All experimental co‐cultures seen in Table 1 were analyzed with three biological replicates. Hence, a total of 6 and 9 nano‐LC‐MS/MS runs were analyzed on both mass spectrometers, resulting in 31 038 and 85 410 MS/MS spectra recorded, respectively.

### 2.3 MS/MS database search and abundance analysis

Compiled MS/MS spectra were searched against the annotated coding domain sequences (CDS) of each strain downloaded from the NCBI (date 20/06/2012). The CDS database of the corresponding co‐cultured *Synechococcus* strain and typical contaminants were included during MS/MS searches in order to avoid false attributions. Experiment 1: searches were carried out with Mascot software (Matrix Science, version 2.2.04) using parameters previously established 15. Mascot results were parsed and peptides were filtered at a *p* value below 0.05. Experiment 2: tandem mass spectra were extracted and analyzed using Mascot (version 2.4.1). Mascot was set up assuming the digestion enzyme trypsin and allowing two miss cleavages with a fragment ion mass tolerance of 0.80 Da and a parent ion tolerance of 20 ppm. Carbamidomethyl of cysteine was specified as a fixed modification and oxidation of methionine as a variable modification. Scaffold (version 4.3.4, Proteome Software Inc.) was used to validate MS/MS‐based peptide and protein identifications. Peptide identifications were accepted if they could be established at greater than 95.0% probability by the Scaffold Local FDR algorithm. Protein identifications with at least two identified peptides were accepted at 95% probability achieving an FDR less than 0.1%. Protein probabilities were assigned by the Protein Prophet algorithm 27. Proteins that contained similar peptides and could not be differentiated based on MS/MS analysis alone were grouped to satisfy the principles of parsimony. Protein quantification by normalized spectral count abundance factor (NSAF) was done as previously described 28.

### 2.4 Protein sequence in silico analysis

The theoretical exoproteomes of the three *Roseobacter* strains were obtained from Christie‐Oleza et al. 15. Local BLASTp analyses were done with the BioEdit BLAST tool v.7.0.5.3 29 using default parameters and an *E*‐value cut‐off <10^−20^. Conserved protein domains and motifs were determined using the Conserved Domains tool at the NCBI (http://www.ncbi.nlm.nih.gov/Structure/cdd/wrpsb.cgi). The BLASTp tool of Roseobase was used for determining protein conservation throughout the clade (http://www.roseobase.org).

## 3 Results and discussion

### 3.1 The *R. pomeroyi* DSS‐3 exoproteome does not show strong variation when co‐cultured with two different *Synechococcus* strains

We analyzed by high‐throughput proteomics the exoproteome of *R. pomeroyi* DSS‐3 when co‐cultured with either *Synechococcus* sp. WH7803 or *Synechococcus* sp. WH8102, assigning 11.4 and 7.6% of the total MS/MS spectra to the *Roseobacter* strain and allowing us to validate 56 and 41 polypeptides (Supporting Information Table 1 and 2), respectively. Approximately 40% of the remaining spectra were assigned to the *Synechococcus* strain present in the co‐culture (data not shown) that correlates nicely with the cell ratio obtained at the end of the experiment (Table 1). Table 2 summarizes the detected *R. pomeroyi* DSS‐3 proteins and their functional category, while more detailed results are shown in Supporting Information Table 3.

**Table 2:** Protein categories found in the exoproteome of *R. pomeroyi* DSS‐3 when in co‐culture with different *Synechococcus* strains

Co‐culture with		
	WH7803^a^^)^	WH8102^a^^)^
Exoenzyme/Interaction	14.0%	6.1%
Motility	9.9%	17.4%
Active transporters	40.3%	40.8%
ROS stress	2.0%	0.0%
Prophage	13.1%	3.1%
Intracellular functions	20.7%	32.6%

a NSAF abundance of the proteins from each category.

Only a small variation in the *R. pomeroyi* DSS‐3 exoproteome occurred when grown in co‐culture with the two different *Synechococcus* strains (Supporting Information Table 3). A lower number of detected proteins was obtained with strain WH8102, possibly due to the lower production of DOM by this more oligotrophic organism (Christie‐Oleza et al. unpublished) 30. The only clear difference was in the class of “intracellular proteins” that were present in the exoproteome, which is easily explained by the random nature of the proteins leaked from cells.

#### 3.1.1 Active transporters and motility proteins

Active ABC and TRAP transporters represented over 40% of the total abundance of exoproteins in the co‐cultures as judged from their NSAF, all 25 detected proteins belonging to the solute‐binding component of the different transport complexes (Supporting Information Table 3). This is not surprising as *Roseobacter* strains are known to encode an unusually large number of these kinds of transporters 31, a fact also consistent with their known abundance in *Roseobacter* exoproteomes 15, 20. In the nutrient‐poor conditions of the co‐cultures *R. pomeroyi* DSS‐3 is likely to accentuate the production of active transporters in order to scavenge different sources of carbon and energy 15, in this case, the diverse pool of DOM produced by *Synechococcus*. An increase in flagellar structural proteins was also a feature of the co‐cultures (9.9 and 17.4% for WH7803 and WH8102, respectively, Table 2), consistent with these potentially starved *R. pomeroyi* DSS‐3 cells searching for new nutrient sources 15.

#### 3.1.2 Exoenzymes and interaction proteins

**Figure 1:**
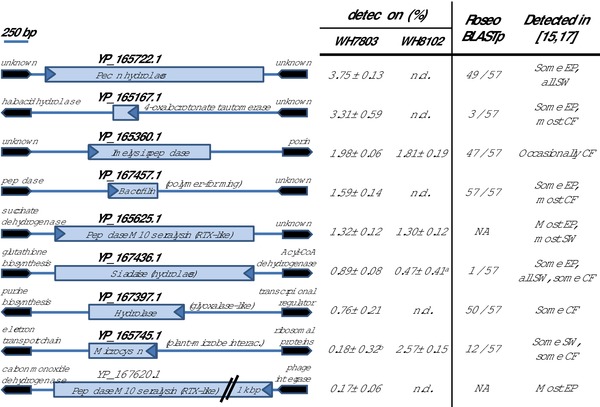
Nine exoenzymes and interaction proteins secreted by *R. pomeroyi* DSS‐3 when co‐cultured with *Synechococcus*. Their annotated function was inferred by the conserved domains they contain. Gene size is represented to scale but flanking regions are not. Arrow point indicates transcription orientation. The relative abundance detected with each *Synechococcus* strain is indicated, n.d. meaning “not detected.” Column “Roseo BLASTp” indicates the number of sequenced *Roseobacter* strains that encode each protein (currently 57 in the Roseobase). NA is indicated for those proteins for which a BLASTp search did not retrieve a result in the Roseobase website. The detection of each protein in previous proteomic analyses (references 15, 17), where cells were pregrown in a similar way and exoproteomes were processed with the same protocol, is indicated as follows: EP, exoproteome analysis in different culture conditions with easily assimilated organic carbon (i.e. marine broth); SW, exoproteomes achieved from incubating *R. pomeroyi* DSS‐3 in natural seawater; CF, cellular fraction proteomic analyses. Superscripts ^a^ and ^b^ represent proteins detected in two and one of the three replicate samples, respectively, whereas all other proteins were detected in the three biological replicates analyzed.

The identification of proteins with potential exoenzyme function, or involved in interacting with *Synechococcus*, were less obvious than previously anticipated as their function was unclear and, hence, poorly annotated. A potential enzymatic function could only be inferred from the presence of various putative structural domains. Figure 1 shows the nine proteins belonging to this functional category that were identified when grown with *Synechococcus*, as well as their abundance in each experiment, genomic context, conservation in other *Roseobacter* strains and whether they were detected in previous exoproteome reports. Remarkably, four of them were found in both co‐cultures. Three of these proteins contained peptidase domains and the other three had hydrolytic‐related functions. Two of the peptidase proteins, YP_165625.1 and YP_167620.1, are RTX‐like toxins 32 and were commonly found in previous exoproteomic analyses of *R. pomeroyi* DSS‐3 15. The third identified peptidase, YP_165360.1, was detected in the presence of both *Synechococcus* strains but not in previous reports (Fig. 1) suggesting some level of specificity to the system analyzed here. Interestingly, the two potential polysaccharide hydrolases YP_165722.1 and YP_167436.1 were previously detected in natural seawater experiments (see 15), meaning these secreted enzymes are likely to be relevant to natural marine environments. While the pectin hydrolase is highly conserved within the *Roseobacter* clade, the sialidase‐like enzyme is strain‐specific (Fig. 1). YP_167457.1 and YP_165745.1 are two proteins with a potential role in interactions. A conserved domain (pfam07484), named “phage tail collar,” found in the latter protein (YP_165745.1), is known to be involved in plant‐microbe interactions 33, 34. This protein was previously detected in natural seawater experiments but only in those where the water was obtained from a eutrophic marina 15. Its role in interacting with photosynthetic primary producers and the underlying molecular mechanism are clearly worth further study.

#### 3.1.3 Gene transfer agents

**Figure 2:**
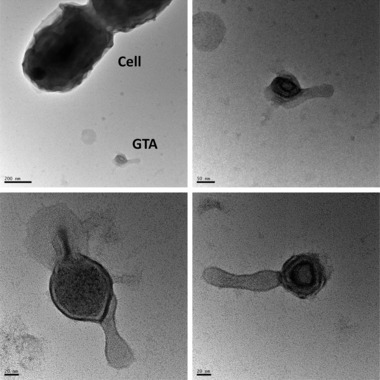
Potential GTAs observed in *Synechococcus* sp WH7803–*Ruegeria pomeroyi* DSS‐3 co‐cultures. Images were taken by transmission electron microscopy after fixing the culture with 2.5% glutaraldehyde and staining with 2% w/v uranium acetate. The size of the GTA is approximately 50 nm.

Remarkably, over 13% of the exoprotein abundance was due to five structural prophage proteins (co‐culture with WH7803 strain, Table 2). Interestingly, these phage‐like structures were previously described as gene transfer agents (GTA) 35, 36. These GTAs are well characterized in *Rhodobacter capsulatus* 37, 38, 39 and are particularly highly conserved among the *Roseobacter* clade 40 (present in 48 of the 57 sequenced genomes). Here, the identification of five different capsid proteins suggests that these GTAs are actively produced and accumulated in the milieu. Furthermore, the major capsid protein YP_167486.1 was also abundantly detected in the coculture with *Synechococcus* sp. WH8102. Images obtained by transmission electron microscopy show the presence in the culture of particles that resemble GTAs previously identified by Biers et al. (Fig. 2) 35. Looking retrospectively at previously reported datasets we found the major capsid protein was also detected in the milieu of stationary phase marine broth cultures and all natural seawater incubations 15. Conversely, they were undetectable in cellular fractions 17, confirming their exoproteomic nature. Further work needs to be performed in order to elucidate the role of the GTA in this particular synthetic community.

#### 3.1.4 PaxA

**Figure 3:**
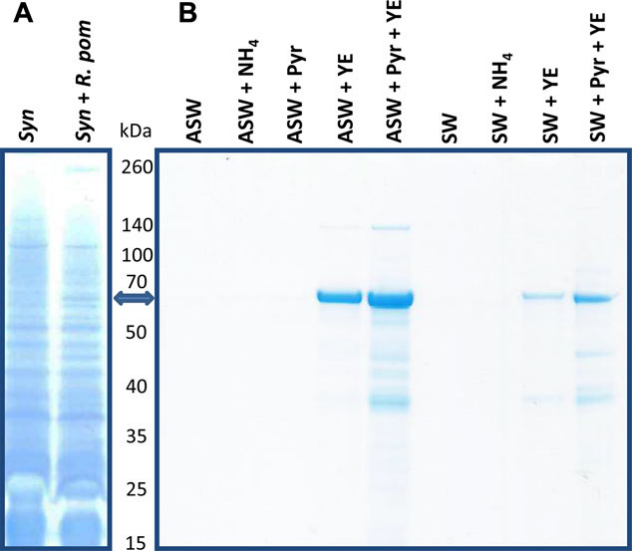
Culture exoproteomes. Concentrated exoproteomes equivalent to 40 mL of culture milieu were resolved by 10% Tris‐Bis NuPAGE gel (Invitrogen) and stained with coomassie G‐250 (SimplyBlue SafeStain, Invitrogen). (A) Exoproteome from an axenic culture of *Synechococcus* sp WH7803 (*Syn*) and when co‐cultured with *R. pomeroyi* DSS‐3 (*Syn* + *R. pom*). (B) Exoproteome of *R. pomeroyi* DSS‐3 when grown with different supplements. ASW, artificial seawater; SW, autoclaved natural seawater; NH_4_, indicates the addition of 5 mM (NH_4_)_2_SO_4_; Pyr, indicates the addition of 0.5% w/v pyruvate; YE, indicates readdition of 0.005% w/v yeast extract. The double arrow indicates the band corresponding to PaxA.

Notable in the co‐cultures was the complete absence of PaxA (YP_165496.1). This RTX‐like toxin represents over 50% of the total exoproteome of *R. pomeroyi* DSS‐3 when grown in optimal laboratory conditions as a stand‐alone culture 15, 20 whereas in these experiments it remained undetected. Similarly, SDS‐PAGE analysis of the *Synechococcus* sp WH7803‐*R. pomeroyi* DSS‐3 co‐culture (Fig. 3A) showed no band of the expected size of PaxA in the exoproteome. We then tested *R. pomeroyi* DSS‐3 in different media to confirm the physiological conditions under which the production of PaxA was repressed and observed that the production of this secreted protein was independent of the presence of a source of carbon and energy or nitrogen (ammonium), but relied instead on the addition of yeast extract (0.005% w/v) as a possible source of vitamins (Fig. 3B). The reason for adding ammonium was that *R. pomeroyi* DSS‐3 cannot use nitrate as a source of nitrogen 31. *R. pomeroyi* DSS‐3 is also known to require vitamin supplements for growth, so during the co‐culture with *Synechococcus* these may be supplied by the photoautotroph but at very small doses that would explain the slow growth rate but still high cell yields (over 5 × 10^8^ cells m/L) the *Roseobacter* strain reaches in this kind of synthetic community. Hence, we consider a mutualistic interaction is taking place within our synthetic community at the time point we sampled the exoproteomes. Interestingly, this is not always the case as mutualistic interactions of *Roseobacter* strains can become antagonistic when grown with different phototrophs 41, 42.

### 3.2 Different *Roseobacter* strains have similar exoproteomes in the presence of the same DOM source but a strain‐specific repertoire of exoenzymes

**Figure 4:**
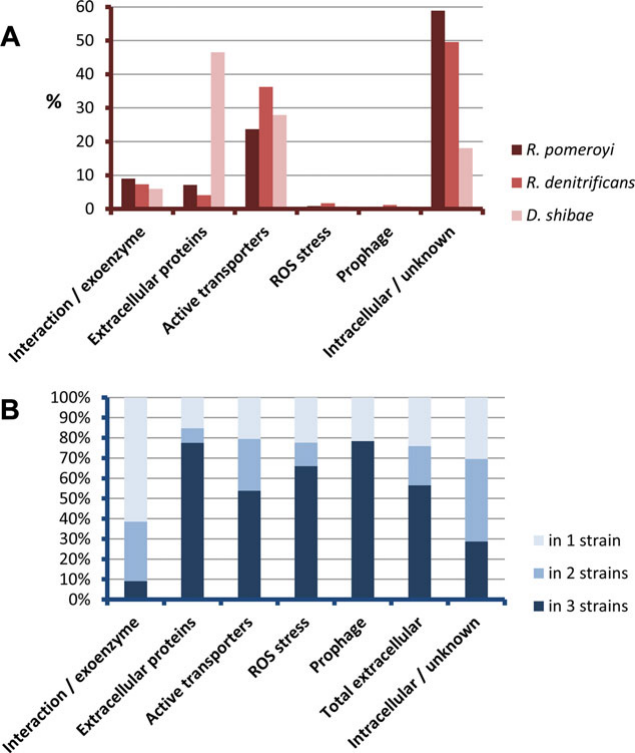
Functional categories of the exoproteomes of three *Roseobacter* strains in terms of abundance. (A) Accumulated NSAF within each functional category documented in Supporting Information Table 5. (B) Hundred percent stacked column chart representing the fraction of each functional category that was detected in one, two, and all strains.

During this second experiment, we achieved a deeper coverage of the exoproteome of three different *Roseobacter* strains (*R. pomeroyi* DSS‐3, *R. denitrificans* OCh114, and *D. shibae* DFL‐12) when cultured with the same source of DOM (i.e. co‐culture with our model strain *Synechococcus* sp. WH7803) by (i) increasing the initial *Roseobacter* cell inoculum (Table 1), (ii) using a faster and more sensitive mass spectrometer (Orbitrap Fusion), and (iii) shortening the incubation time, to decrease the number of proteins leaked by *Synechococcus* that tended to mask those produced by the *Roseobacter* strain. A total of 3802, 3188, and 1663 MS/MS spectra from biological triplicate experiments were assigned to each *Roseobacter* strain allowing us to validate 288, 206, and 96 different polypeptides, respectively (Supporting Information Table 4). All detected proteins were grouped by homologous clusters among the three strains and classified by functional categories (Supporting Information Table 5) to obtain a comparative analysis between strains. Figure 4 shows the proteomic results per functional category. Figure 4A represents the detected abundance of each category in all three strains.

#### 3.2.1 Exoenzymes and interaction clusters

It is interesting to note that on this occasion we detected 42 potential exoenzyme/interaction protein clusters that represented 6–9% of the *Roseobacter* exoproteome in terms of abundance (Fig. 4A). Twelve of the 42 clusters were RTX‐related proteins containing peptidase domains (Supporting Information Table 5). PaxA was detected but in low abundance (2.3%; much lower than when grown in rich media, when it represented 50% of the exoproteome [see 15, 20]), possibly from the remains of cells previously grown in marine broth. Again, most of the listed exoenzymes had no clear and defined function, and were only identified through their catalytic domains. Hence, this list of secreted enzymes points to a general lack of knowledge on the hydrolytic reactions of marine exoproteins.

#### 3.2.2 Extracellular proteins

Proteins classified as extracellular proteins were those involved in motility (flagellar proteins), porins, or peptidoglycan/outer membrane‐related proteins. Surprisingly, flagellar proteins were especially abundant in *D. shibae* DFL‐12 following co‐culture with *Synechococcus* sp. WH7803, detecting a total of eight different components of the flagellar structure that represented over 43.5% of the total exoproteome (Fig. 4A). The flagellin protein YP_168655.1 represented 37.5% of the global proteome. These motility proteins had previously been shown to have an important role in the lifestyle of *D. shibae* DFL‐12 15 but had not been reported in such high abundance.

#### 3.2.3 Active transporters

As previously seen, membrane and substrate‐binding components of active transporters represented an important fraction of the exoproteome in all three *Roseobacter* strains (23.7, 36.2, and 27.9%, Fig. 4A) detecting a total of 92 homology clusters (Supporting Information Table 5). Most of these were predicted to transport protein sub‐products (amino acids and oligopeptides) and carbohydrates (sugars and C4‐dicarboxylates), with 26 homology clusters each. In terms of detected abundance, while *R. pomeroyi* DSS‐3 showed a preference for amino acid and peptide transporters (10.3% total NSAF) when compared to carbohydates (2.3%), *R. denitrificans* OCh114, and *D. shibae* DFL‐12 showed a more balanced behavior (9% versus 10.2% and 7.1% versus 5.9%, respectively). Furthermore, transporters for other relevant substrates were also detected, i.e. amines, putrescine, taurine, sulphate, phosphates, and iron (Supporting Information Table 5).

#### 3.2.4 Other relevant proteins

Those proteins involved in dealing with oxidative stress are not abundant in the different exoproteomes but have important biological significance. It has been reported that the “helping” effect heterotrophs have in picocyanobacterial cultures is related to scavenging reactive oxygen species 43. In our exoproteomic survey we detected superoxide dismutase and catalase/peroxidase, both suggested to possess a nonclassical secretion by in silico predictions. The prophage‐like GTA was also experimentally detected in all three *Roseobacter* exoproteomes proving commonality of this mechanism for maintaining genetic exchange in this group of bacteria.

When considering the entire dataset of homologous clusters we can conclude that the secreted fraction of the three different *Roseobacter* strains is similar when grown with the same source of DOM. Hence, when considering only the secreted fraction, over 56% of the exoproteome comprises proteins detected in all three strains and 76% was detected in at least two strains (Fig. 4B). Nevertheless, this general trend is not observed in the “exoenzyme/interaction” fraction as only 9% of this category is common to the three strains while 38% are shared by two strains (Fig. 4B). From the 42 homologous clusters of secreted enzymes and interaction proteins, only one was detected in all three strains (a highly conserved secreted nucleotidase) and nine in two strains (Supporting Information Table 5). The other 32 detected clusters were specifically detected in only one strain highlighting the uniqueness of each heterotroph in terms of its exoenzyme and interaction repertoire and, ultimately, the potential for niche partitioning due to substrate and interaction specialization. The percentage of protein sequence similarities is also lower in the intracellular fraction but, as previously highlighted, this is expected due to the randomness of leaked proteins.

## 4 Concluding remarks

Microorganisms are a crucial component of the majority of Earth's ecosystems so the development of new technical approaches such as next‐generation sequencing and high‐throughput proteomics makes it entirely feasible to study these microbiomes in unprecedented new detail. Because microbes are never alone in nature, by combining specific individuals in the laboratory we can mimic natural microbiomes and, in so doing, understand real microbial dynamics. Such approaches are likely to become increasingly more popular in the coming years. Here, we used two‐microbe model systems in order to study how a relevant marine heterotroph, i.e. *Roseobacter*, faces a more realistic source of carbon and energy such as that produced or leaked by a marine photosynthetic primary producer, i.e. the relevant picocyanobacteria *Synechococcus*. In this close to reality situation, the heterotroph produced an interesting repertoire of secreted proteins, either for interaction purposes or for hydrolyzing large biopolymers for subsequent remineralization and assimilation. Furthermore, while these secreted proteins, such as those involved in active transport, had similar patterns in the different *Roseobacter* strains, those involved in interaction or exocatalytic functions showed a much higher variability between strains. Hence, it is probable that this strain‐specific repertoire allows closely related microorganisms to target slightly different substrates that coexist in natural environments, which is likely an important facet of the niche partitioning process. Certainly, the fact that the majority of the enzymes we identified had no characterized function, suggests the strategy we present here will be an important stepping stone for flagging ecologically relevant biopolymer‐hydrolyzing exoenzymes that require further biochemical analysis.

## Supporting information

As a service to our authors and readers, this journal provides supporting information supplied by the authors. Such materials are peer reviewed and may be re‐organized for online delivery, but are not copy‐edited or typeset. Technical support issues arising from supporting information (other than missing files) should be addressed to the authors.

Table S1: List of detected peptides and polypeptides belonging to Ruegeria pomeroyi DSS‐3 when grown in the presence of Synechococcus sp WH7803.

Table S2: List of detected peptides and polypeptides belonging to Ruegeria pomeroyi DSS‐3 when grown in the presence of Synechococcus sp WH8102.

Table S3: Functional groups of polypeptides belonging to Ruegeria pomeroyi DSS‐3 detected when independently co‐cultured with Synechococcus sp. WH7803 and WH8102.

Table S4: List of detected peptides and polypeptides belonging to three different Roseobacter strains when grown in the presence of Synechococcus sp WH7803.

Table S5: Homologous clusters for comparative analysis of the exoproteomes of the three studied Roseobacter strains when in co‐culture with Synechococcus sp. WH7803.

## References

[1] Falkowski, P. , Ocean science: the power of plankton. Nature 2012, 483, S17–S20. 22378122 10.1038/483S17a

[2] Biers, E. J. , Sun, S. , Howard, E. C. , Prokaryotic genomes and diversity in surface ocean waters: interrogating the global ocean sampling metagenome. Appl. Environ. Microbiol. 2009, 75, 2221–2229. 19201952 10.1128/AEM.02118-08 PMC2663191

[3] Cuvelier, M. L. , Allen, A. E. , Monier, A. , McCrow, J. P. et al., Targeted metagenomics and ecology of globally important uncultured eukaryotic phytoplankton. Proc. Natl. Acad. Sci. USA 2010, 107, 14679–14684. 20668244 10.1073/pnas.1001665107 PMC2930470

[4] Jardillier, L. , Zubkov, M. V. , Pearman, J. , Scanlan, D. J. , Significant CO2 fixation by small prymnesiophytes in the subtropical and tropical northeast Atlantic Ocean. ISME J. 2010, 4, 1180–1192. 20393575 10.1038/ismej.2010.36

[5] Breitbart, M. , Middelboe, M. , Rohwer, F. , in: Kirchman D. L. (Ed.), Microbial Ecology of the Oceans, Wiley‐Blackwell, NJ 2008, pp. 443–479.

[6] Jürgens, K. , Massana, R. , in: Kirchman D. L. (Ed.), Microbial Ecology of the Oceans, Wiley‐Blackwell, NJ 2008, pp. 383–441.

[7] Nagata, T. , in: Kirchman D. L. (Ed.), Microbial Ecology of the Oceans, Wiley‐Blackwell, NJ 2008, pp. 207–242.

[8] Azam, F. , Microbial control of oceanic carbon flux: the plot thickens. Science 1998, 280, 694–696.

[9] Pedler, B. E. , Aluwihare, L. I. , Azam, F. , Single bacterial strain capable of significant contribution to carbon cycling in the surface ocean. Proc. Natl. Acad. Sci. USA 2014, 111, 7202–7207. 24733921 10.1073/pnas.1401887111 PMC4034236

[10] Weiss, M. S. , Abele, U. , Weckesser, J. , Welte, W. et al., Molecular architecture and electrostatic properties of a bacterial porin. Science 1991, 254, 1627–1630. 1721242 10.1126/science.1721242

[11] Vetter, Y. A. , Deming, J. W. , Growth rates of marine bacterial isolates on particulate organic substrates solubilized by freely released extracellular enzymes. Microb. Ecol. 1999, 37, 86–94. 9929397 10.1007/s002489900133

[12] D' Ambrosio, L. , Ziervogel, K. , MacGregor, B. , Teske, A. , Arnosti, C. , Composition and enzymatic function of particle‐associated and free‐living bacteria: a coastal/offshore comparison. ISME J. 2014, 8, 2167–2179. 24763371 10.1038/ismej.2014.67 PMC4992077

[13] Arnosti, C. , Microbial extracellular enzymes and the marine carbon cycle. Ann. Rev. Mar. Sci. 2011, 3, 401–425. 10.1146/annurev-marine-120709-142731 21329211

[14] Baltar, F. , Aristegui, J. , Gasol, J. M. , Lekunberri, I. , Herndl, G. J. , Mesoscale eddies: hotspots of prokaryotic activity and differential community structure in the ocean. ISME J. 2010, 4, 975–988. 20357833 10.1038/ismej.2010.33

[15] Christie‐Oleza, J. A. , Pina‐Villalonga, J. M. , Bosch, R. , Nogales, B. , Armengaud, J. , Comparative proteogenomics of twelve *Roseobacter* exoproteomes reveals different adaptive strategies among these marine bacteria. Mol. Cell. Proteomics 2012, 11, M111 013110. 22122883 10.1074/mcp.M111.013110 PMC3277765

[16] Armengaud, J. , Christie‐Oleza, J. A. , Clair, G. , Malard, V. , Duport, C. , Exoproteomics: exploring the world around biological systems. Expert. Rev. Proteomics 2012, 9, 561–575. 23194272 10.1586/epr.12.52

[17] Christie‐Oleza, J. A. , Fernandez, B. , Nogales, B. , Bosch, R. , Armengaud, J. , Proteomic insights into the lifestyle of an environmentally relevant marine bacterium. ISME J. 2012, 6, 124–135. 21776030 10.1038/ismej.2011.86 PMC3246242

[18] Kossmehl, S. , Wohlbrand, L. , Druppel, K. , Feenders, C. et al., Subcellular protein localization (cell envelope) in *Phaeobacter inhibens* DSM 17395. Proteomics 2013, 13, 2743–2760. 23907795 10.1002/pmic.201300112

[19] Zech, H. , Hensler, M. , Kossmehl, S. , Druppel, K. et al., Adaptation of *Phaeobacter inhibens* DSM 17395 to growth with complex nutrients. Proteomics 2013, 13, 2851–2868. 23613352 10.1002/pmic.201200513

[20] Christie‐Oleza, J. A. , Armengaud, J. , In‐depth analysis of exoproteomes from marine bacteria by shotgun liquid chromatography‐tandem mass spectrometry: the *Ruegeria pomeroyi* DSS‐3 case‐study. Mar. Drugs 2010, 8, 2223–2239. 20948905 10.3390/md8082223 PMC2953401

[21] Durighello, E. , Christie‐Oleza, J. A. , Armengaud, J. , Assessing the exoproteome of marine bacteria, lesson from a RTX‐toxin abundantly secreted by *Phaeobacter strain* DSM 17395. PloS One 2014, 9, e89691. 24586966 10.1371/journal.pone.0089691 PMC3933643

[22] Christie‐Oleza, J. A. , Armengaud, J. , Guerin, P. , Scanlan, D. J. , Functional distinctness in the exoproteomes of marine *Synechococcus* . Environ. Microbiol. (in press). doi:10.1111/1462-2920.12822 10.1111/1462-2920.12822 PMC4949707 25727668

[23] Biller, S. J. , Schubotz, F. , Roggensack, S. E. , Thompson, A. W. et al., Bacterial vesicles in marine ecosystems. Science 2014, 343, 183–186. 24408433 10.1126/science.1243457

[24] Wilson, W. H. , Carr, N. G. , Mann, N. H. , The effect of phosphate status on the kinetics of cyanophage infection in the oceanic cyanobacterium *Synechococcus* sp. WH7803. J. Phycol. 1996 32, 506–516.

[25] Clair, G. , Roussi, S. , Armengaud, J. , Duport, C. , Expanding the known repertoire of virulence factors produced by *Bacillus cereus* through early secretome profiling in three redox conditions. Mol. Cell. Proteomics 2010, 9, 1486–1498. 20368289 10.1074/mcp.M000027-MCP201 PMC2938089

[26] de Groot, A. , Dulermo, R. , Ortet, P. , Blanchard, L. et al., Alliance of proteomics and genomics to unravel the specificities of Sahara bacterium *Deinococcus deserti* . PLoS Genet. 2009, 5, e1000434. 19370165 10.1371/journal.pgen.1000434 PMC2669436

[27] Nesvizhskii, A. I. , Keller, A. , Kolker, E. , Aebersold, R. , A statistical model for identifying proteins by tandem mass spectrometry. Anal. Chem. 2003, 75, 4646–4658. 14632076 10.1021/ac0341261

[28] Liu, H. , Sadygov, R. G. , Yates, J. R. , 3rd, A model for random sampling and estimation of relative protein abundance in shotgun proteomics. Anal. Chem. 2004, 76, 4193–4201. 15253663 10.1021/ac0498563

[29] Hall, T. A. , BioEdit: a user‐friendly biological sequence alignment editor and analysis program for Windows 95/98/NT. Nucl. Acids Symp. Ser. 1999, 41, 95–98.

[30] Scanlan, D. J. , Ostrowski, M. , Mazard, S. , Dufresne, A. et al., Ecological genomics of marine picocyanobacteria. Microbiol. Mol. Biol. Rev. 2009, 73, 249–299. 19487728 10.1128/MMBR.00035-08 PMC2698417

[31] Moran, M. A. , Buchan, A. , Gonzalez, J. M. , Heidelberg, J. F. et al., Genome sequence of *Silicibacter pomeroyi* reveals adaptations to the marine environment. Nature 2004, 432, 910–913. 15602564 10.1038/nature03170

[32] Linhartova, I. , Bumba, L. , Masin, J. , Basler, M. et al., RTX proteins: a highly diverse family secreted by a common mechanism. FEMS Microbiol. Rev. 2010, 34, 1076–1112. 20528947 10.1111/j.1574-6976.2010.00231.x PMC3034196

[33] Cubo, M. T. , Economou, A. , Murphy, G. , Johnston, A. W. , Downie, J. A. , Molecular characterization and regulation of the rhizosphere‐expressed genes *rhiABCR* that can influence nodulation by *Rhizobium leguminosarum* biovar viciae. J. Bacteriol. 1992, 174, 4026–4035. 1597418 10.1128/jb.174.12.4026-4035.1992 PMC206112

[34] Thomassen, E. , Gielen, G. , Schutz, M. , Schoehn, G. et al., The structure of the receptor‐binding domain of the bacteriophage T4 short tail fibre reveals a knitted trimeric metal‐binding fold. J. Mol. Biol. 2003, 331, 361–373. 12888344 10.1016/s0022-2836(03)00755-1

[35] Biers, E. J. , Wang, K. , Pennington, C. , Belas, R. et al., Occurrence and expression of gene transfer agent genes in marine bacterioplankton. Appl. Environ. Microbiol. 2008, 74, 2933–2939. 18359833 10.1128/AEM.02129-07 PMC2394915

[36] Lang, A. S. , Zhaxybayeva, O. , Beatty, J. T. , Gene transfer agents: phage‐like elements of genetic exchange. Nat. Rev. Microbiol. 2012, 10, 472–482. 22683880 10.1038/nrmicro2802 PMC3626599

[37] Hynes, A. P. , Mercer, R. G. , Watton, D. E. , Buckley, C. B. , Lang, A. S. , DNA packaging bias and differential expression of gene transfer agent genes within a population during production and release of the *Rhodobacter capsulatus* gene transfer agent, RcGTA. Mol. Microbiol. 2012, 85, 314–325. 22640804 10.1111/j.1365-2958.2012.08113.x

[38] McDaniel, L. D. , Young, E. C. , Ritchie, K. B. , Paul, J. H. , Environmental factors influencing gene transfer agent (GTA) mediated transduction in the subtropical ocean. PLoS One 2012, 7, e43506. 22905268 10.1371/journal.pone.0043506 PMC3419701

[39] Brimacombe, C. A. , Ding, H. , Beatty, J. T. , *Rhodobacter capsulatus* DprA is essential for RecA‐mediated gene transfer agent (RcGTA) recipient capability regulated by quorum‐sensing and the CtrA response regulator. Mol. Microbiol. 2014, 92, 1260–1278. 24784901 10.1111/mmi.12628

[40] Zhao, Y. , Wang, K. , Budinoff, C. , Buchan, A. et al., Gene transfer agent (GTA) genes reveal diverse and dynamic *Roseobacter* and *Rhodobacter* populations in the Chesapeake Bay. ISME J. 2009, 3, 364–373. 19020557 10.1038/ismej.2008.115

[41] Seyedsayamdost, M. R. , Case, R. J. , Kolter, R. , Clardy, J. , The Jekyll‐and‐Hyde chemistry of *Phaeobacter gallaeciensis* . Nat. Chem. 2011, 3, 331–335. 21430694 10.1038/nchem.1002 PMC3376411

[42] Wang, H. , Tomasch, J. , Jarek, M. , Wagner‐Dobler, I. , A dual‐species co‐cultivation system to study the interactions between *Roseobacters* and dinoflagellates. Front. Microbiol. 2014, 5, 311. 25009539 10.3389/fmicb.2014.00311 PMC4069834

[43] Morris, J. J. , Johnson, Z. I. , Szul, M. J. , Keller, M. , Zinser, E. R. , Dependence of the cyanobacterium *Prochlorococcus* on hydrogen peroxide scavenging microbes for growth at the ocean's surface. PloS One 2011, 6, e16805. 21304826 10.1371/journal.pone.0016805 PMC3033426

